# Experimental Study on Shear-Peeling Debonding Behavior of BFRP Sheet-to-Steel Interfaces

**DOI:** 10.3390/polym15092216

**Published:** 2023-05-08

**Authors:** Hanyang Xue, Dafu Cao, Zhanzhan Tang, Qing Liu, Siji Zhu, Jiaqi Liu, Chuanzhi Sun

**Affiliations:** 1 Department of Civil Engineering, College of Civil Science and Engineering, Yangzhou University, Yangzhou 225127, Chinamz120211015@stu.yzu.edu.cn (Q.L.);; 2 Jiangsu Province Engineering Research Center of Prefabricated Building and Intelligent Construction, Suqian 223800, China

**Keywords:** BFRP, steel, debonding, bond strength, shear-peeling failure, mixed-mode

## Abstract

In order to study the failure mode and debonding behavior of the interface between BFRP (basalt fiber reinforced polymer) sheet and structural steel under mixed-mode loading conditions, eighteen specimens with different initial angles were tested in this study. The specimens were designed with different initial angles to ensure that the interface performed under mixed-mode loading conditions. The relations between the bond strengths, failure modes, and initial angles were investigated. A new evaluation method to predict the interfacial bond strength under shear-peeling loading mode was proposed. The test results show that specimens with a smaller initial angle are more likely to exhibit a shear debonding failure at the interface between the steel plate and adhesive. In contrast, specimens with a larger initial angle are more likely to exhibit peeling of the interface. The ultimate tensile strength of the specimen is higher with a smaller initial angle. The results predicted by the proposed method are in good agreement with the experimental results.

## 1. Introduction

Steel structures have many advantages, including excellent seismic performance, high strength, fast construction speed, stable quality, light weight, and green environmental protection [1,2,3,4]. However, due to the external environment as well as internal defects and human factors, steel structures are likely to accumulate damage over the course of their service life. Although traditional welding, bolted connections, and external steel plate reinforcement methods can improve the bearing capacity of the damaged structures, they may also cause further damage and additional weight to the structures [5,6,7,8,9]. There are many advantages to using fiber-reinforced polymer sheets for reinforcement, such as corrosion resistance, light weight, simple construction, environmental friendliness, etc., which can be adopted to improve the bearing strength, corrosion resistance, and compressive buckling capacity of the steel structures [10,11,12,13,14].

The advantages of fiber-reinforced polymer (FRP) include corrosion resistance, light weight, simplicity of construction, no pollution, and superior fatigue resistance. Steel structures can be strengthened using FRP to increase their ultimate strength and buckling resistance, as well as protect them from corrosion [15,16,17,18]. At present, the main fiber-reinforced polymers are carbon-fiber-reinforced polymer (CFRP), glass-fiber-reinforced polymer (GFRP), and basalt-fiber-reinforced polymer (BFRP). Basalt fiber is a pure, natural, inorganic material made from natural rocks. After use, it can be returned to nature without special treatment. In comparison with CFRP and GFRP, BFRP has the advantages of environmental protection, low cost, superior insulation, low hygroscopicity, and abundant raw materials [19,20].

Many researchers have carried out experiments on the bonding properties of FRP-to-steel interface, and corresponding theoretical formulae have been proposed [21,22,23,24,25]. Doroudi et al. [26] investigated the behavior of CFRP-to-steel bonded joints under quasi-static cyclic loading using nine single shear pull-off test specimens, in which three different bond thicknesses and both quasi-static monotonic and quasi-static cyclic loading methods were considered. The results showed that the stiffness of the load-displacement curves tends to decrease with an increasing number of loading cycles. Bocciarelli et al. [27] conducted an experimental study on the fatigue behavior of steel structures retrofitted by FRP materials. The results showed that the fatigue resistance of the interface between the steel plate and the CFRP plate is remarkably superior to that of welded cover plates. Dehghani et al. [28] proposed a new method for the analysis of bonded connections of CFRP and steel substrates. A comparison of results obtained from their proposed model and experimental data showed that the ultimate debonding load could be accurately estimated by their proposed model. In this model, unlike some previous bond-slip models, the ultimate debonding load is independent of the thickness of the adhesive. Biscaia et al. [29] investigated the performance of CFRP-to-steel bonded joints under different temperatures (20 °C, 35 °C, 50 °C, 65 °C, 80 °C, and 95 °C). According to the local bond-slip behavior of the tested specimens, a temperature-dependent bond-slip model with a bi-linear shape was proposed and implemented into commercial software based on the finite element method. Yu et al. [30] conducted an experimental study on the behavior of CFRP-to-steel bonded interfaces by testing a series of single-lap bonded joints. The test results showed that the bond strength of such bonded joints depends strongly on the interfacial fracture energy among other factors. Wu et al. [31] conducted a series of static and fatigue tests on UHM (ultra-high modulus) CFRP plate and steel plate double strap joints to investigate the effect of residual bond strength and residual bond stiffness on bond behavior. Wu et al. [32] studied the bond characteristics between ultra-high modulus (UHM) CFRP laminates with a modulus of 460 GPa and steel through a series of experiments with double strap steel joints bonded with UHM CFRP laminates. The effect of two types of adhesives (Araldite and Sikadur) on the failure modes, bond strength, effective bond length, CFRP strain distribution, adhesive layer shear stress distribution, and bond-slip relationship were discussed. Then, theoretical models were employed for the prediction of the specimen bond strength and effective bond length, and their applicability for UHM CFRP steel joints was verified by comparisons with experimental results. Golewski [33] studied the tensile behavior of double- and triple-adhesive single-lap joints. It was found that the energy required to damage the joint when using double-sided adhesive tape is several times greater than that required to damage a joint made with epoxy. Jawdhari et al. [34] investigated the bond-slip relationship between CFRP rod panels and concrete. They found that the debonding load of a CFRP rod panel is higher than that of an externally bonded conventional CFRP plate of similar cross-sectional area and mechanical properties. Mukhtar et al. [35] tested the adhesive bond between FRP laminate and concrete in both double shear and mixed mode (shear/peeling) using a novel test apparatus. Test results showed that the bond capacity decreases as the peel angle increases. Altaee et al. [36] studied the bond-slip relations of CFRP-steel joints using a new bilinear model. The proposed model resulted in excellent predictions in terms of the ultimate strength, failure mode, and all other interfacial properties. Jawdhari et al. [37] carried out a three-dimensional nonlinear finite element analysis to study the strip panels’ interfacial and flexural properties. As a result, strip panels with finger joints were found to carry more load for a given CFRP thickness and splice plate length.

Most of the above studies focused on the bonding behavior of the interfaces regarding shear forces. However, the failure of the FRP-to-steel interface is related to the mixed-mode debonding failure, i.e., the combined actions of shear and peeling forces [38,39,40]. In order to study the failure mode and debonding behavior of the interface between BFRP (basalt fiber reinforced polymer) sheet and structural steel under mixed-mode loading conditions, eighteen specimens with different initial angles were tested in this study. The specimens were designed with different initial angles to ensure that the interface was tested under shear and peeling mixed-mode conditions. Finally, a new calculation method for predicting the bond strength between BFRP sheets and steel under shear-peeling fracture is proposed.

## 2. Experimental Program

### 2.1. Specimen Design

**Figure 1:**
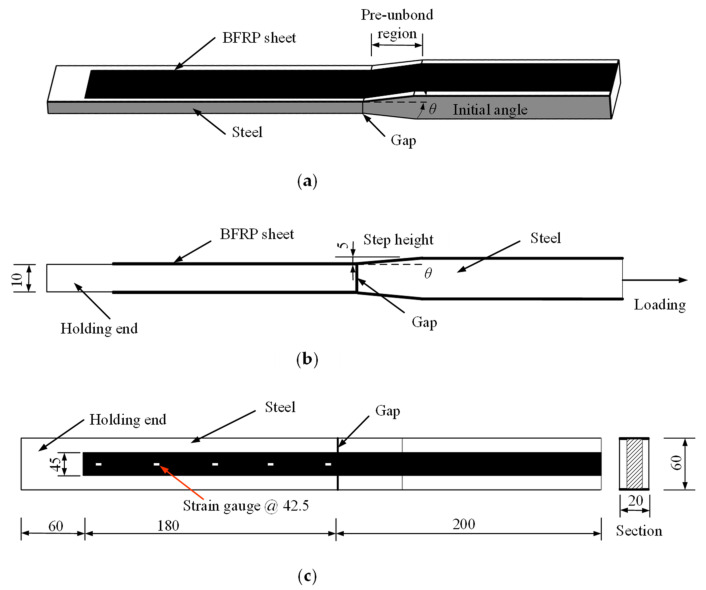
Dimensions and details of test specimens (units: mm): (**a**) Stereogram; (**b**) Elevation; (**c**) Plan.

A total of 18 specimens with BFRP sheets bonded on two sides were prepared and tested, marked as S1 to S9, respectively. Each specimen was composed of two steel plates with a 5 mm length gap between them. Q345qD-grade steel was used for all specimens. The geometric dimensions and details of the specimens are shown in Figure 1. The dimensions of the two steel plates were 240 × 60 × 10 mm (length × width × thickness) and 200 × 60 × 20 mm (length × width × thickness), respectively. Two different cross-sections of steel plates were prepared to allow for the attachment of an initial angle. The initial angle of the steel plates was machined using a computer numerical control (CNC) machine. By adjusting the length of the pre-unbond region of the plate, the initial angle was manufactured without affecting the step height and the angle value of the steel plate. The initial angles were set as 0°, 2°, 4°, 6°, 8°, 10°, 12°, 15° and 20° corresponding to specimens S1 to S9, respectively. The bi-directional fabrics with a nominal thickness of 0.20 mm were produced by Tianlong Company (Jiangsu, China). The areal density of the commercial BFRP composites is 210 g/m^2^, and a commercial two-component epoxy adhesive was used as the polymer matrix. The width and length of the BFRP sheets applied in the current experiment were 45.0 mm and 380 mm, respectively. The specimens were prepared with BFRP sheets bonded on both sides of the steel plates. The BFRP sheet was first bonded to the steel plate on one side, and then to the other side within 15 min. One end of the specimen was clipped with an MTS servo-hydraulic testing machine to make sure the interfacial debonding was triggered on the opposite end. Table 1 shows the design parameters of the tested specimens. Two specimens were tested in each load case.

The specimens were prepared according to the following procedure [33]: (1) The adhesive was a two-component modified epoxy resin with a 4:1 weight ratio between glue A and glue B, and it was stirred at 300 rotations per minute for three minutes before application. (2) The surfaces of the steel plates were sandblasted and cleaned with acetone to ensure good bonding between the BFRP sheets and the steel plates. (3) The surfaces of the BFRP sheets were cleaned with acetone before bonding. (4) The adhesive was placed evenly on the surface of the treated steel plates, and the BFRP sheet was pressed to remove excess adhesive and ensure that no air voids existed at the interface. (5) The system was applied to one side, then flipped to the other side, and the operation was repeated. This method ensured that both sides were completed with the same amount of care and attention to detail. (6) There was a 15 min delay between the first and second applications. (7) The specimens with the adhesive were cured for a minimum of seven days at room temperature before testing.

### 2.2. Material Properties

The adhesive used to bond the BFRP sheet to the steel plate surface was a two-component epoxy adhesive, whose properties are listed in Table 2. The material properties of the adhesive used in the test were provided by the manufacturer. The material properties of the steel plates and the BFRP sheets were tested according to the specifications of GB/T 228.1-2021 [41] and GB/T 3354-1999 [42], respectively. The test results are summarized in Table 3 and Table 4.

### 2.3. Experimental Setup

**Figure 2:**
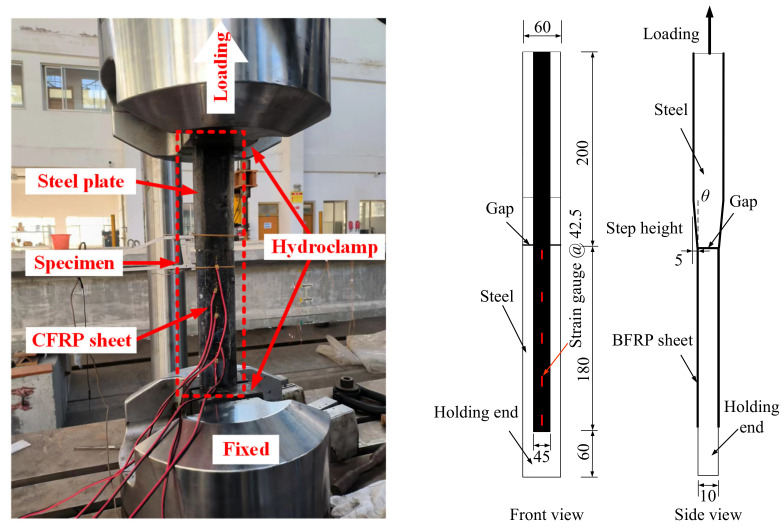
Experimental setup and measurement details.

The experimental setup and details of the deformation measurement of the specimens are shown in Figure 2. Specimen S1 with an initial angle of 0° was selected as the control specimen for comparison, and a shear debonding failure mode was observed in the tests. Specimens S2 to S9 were subjected to a tensile force to cause a shear-peeling debonding failure at the interface. The specimens were fixed at both ends and then tensioned to failure on a 500 kN capacity MTS servo-hydraulic testing machine at a loading speed of 0.1 mm/min [43]. Each specimen was instrumented with five strain gauges on the BFRP sheet surface on one side. These strain gauges were spaced at intervals of 42.5 mm from the middle gap between the steel plates to the fixed end. Figure 1c shows the locations of the five strain gauges. The sensitivity coefficient of these strain gauges was 2.0 ± 1.0%. The strain gauges on the specimen were numbered Y1 to Y5, respectively.

## 3. Results and Discussions

### 3.1. Experimental Phenomena and Failure Modes

**Figure 3:**
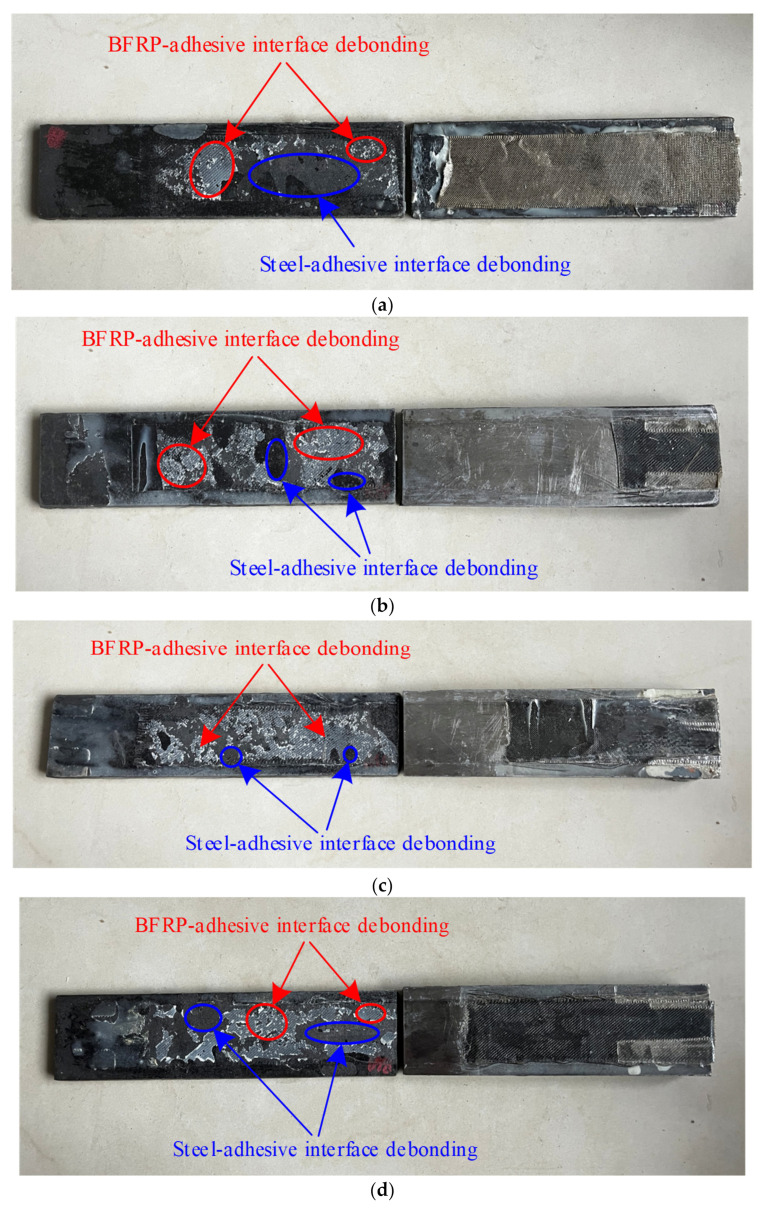

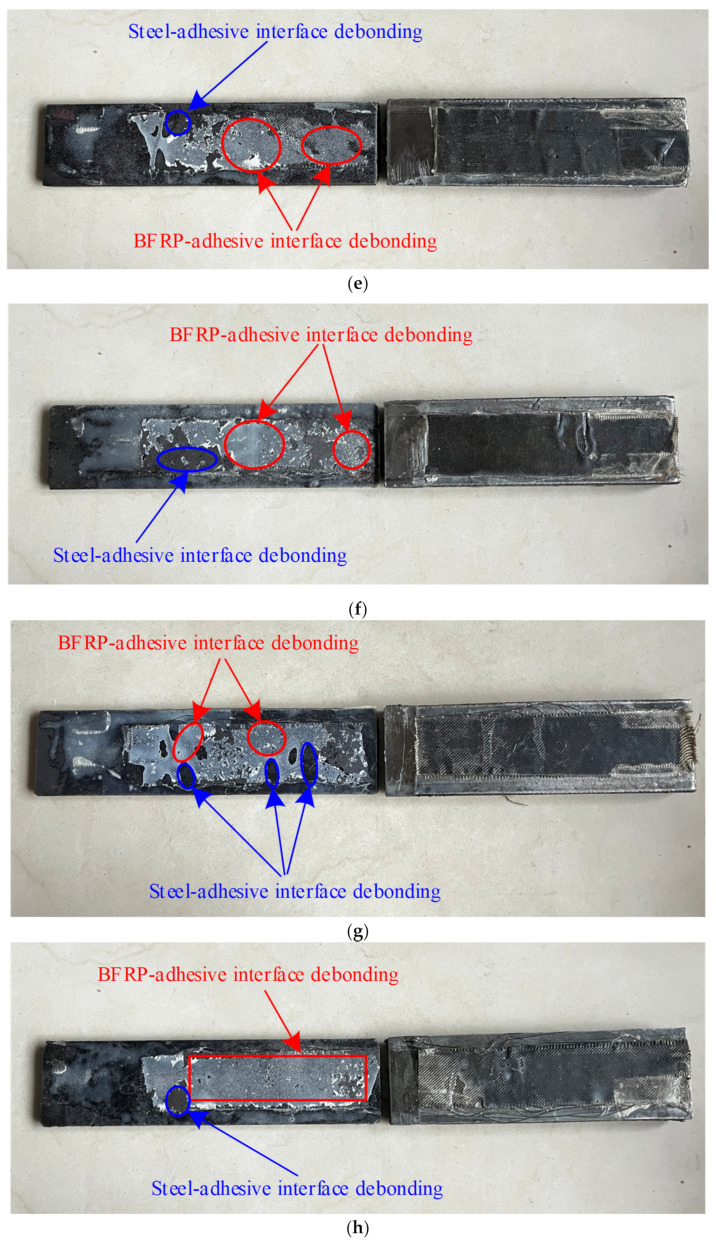

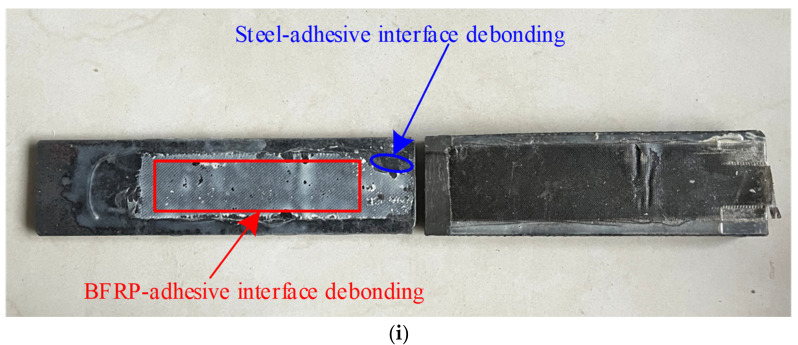
Failure modes of tested specimens: (**a**) S1; (**b**) S2; (**c**) S3; (**d**) S4; (**e**) S5; (**f**) S6; (**g**) S7; (**h**) S8; (**i**) S9.

The failure modes of the tested specimens are shown in Figure 3. The calculated bond strength was determined using the Liu model [44]. All specimens were subjected to a tensile force until debonding failure occurred on either side. At the initial stage of loading, the load increased roughly linearly with the increase in the relative displacement at the interface. As the load continued to increase, the BFRP sheet debonded from the steel surface at the middle gap of the specimen. Subsequently, the debonding of the BFRP sheet gradually expanded to the end of the specimens. Specimen S1 failed in a pure shear debonding mode, while mixed shear and peeling failure modes occurred in specimens S2 to S9. The failure surfaces of all specimens were failure modes of a combination of BFRP-sheet–adhesive failure and steel-plate–adhesive failure. As the loading increased, the debonding transferred gradually from the steel–adhesive interface to the BFRP-sheet–adhesive interface. In other words, the specimens with a smaller initial angle are more likely to exhibit interfacial debonding between the steel plate and the adhesive. In contrast, the specimens with a larger initial angle are more likely to exhibit peeling at the interface between the BFRP sheet and the adhesive. Nevertheless, for specimens with an initial angle, the debonding progress was caused by a combination of two fracture modes, i.e., peeling and shear debonding modes. The normal force at the interface between BFRP sheets and steel plates increases with the increase in the initial angle. When the initial angle of steel plates is greater than 6°, the specimens are more likely to exhibit peeling at the interface between the BFRP sheet and the adhesive.

Table 5 lists the experimental ultimate load (*P*) and the calculated ultimate load (*P*_cal_) of the specimens. It can be seen that the ultimate load of the specimen decreases with the increase in the initial angle. The initial angle has a significant influence on the interfacial bonding behavior of the specimens. The accuracy of the predicted strength by the Liu model is reduced gradually with the increase in the angles since the effect of mixed-mode debonding is not considered.

### 3.2. Load-Displacement Curves

**Figure 4:**
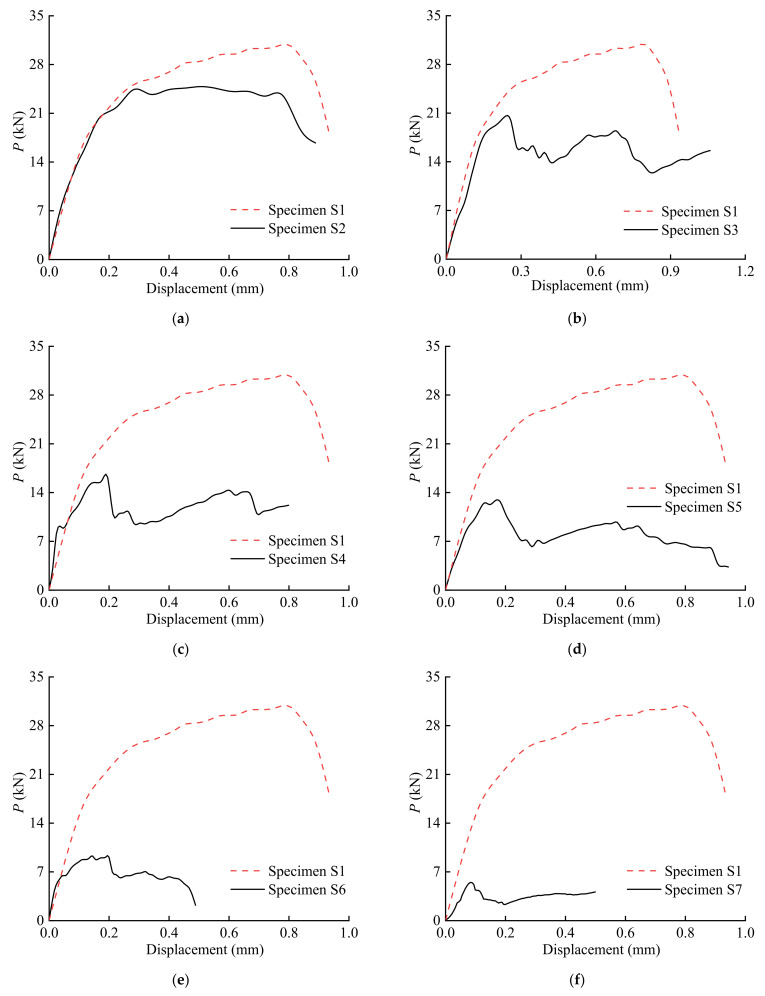

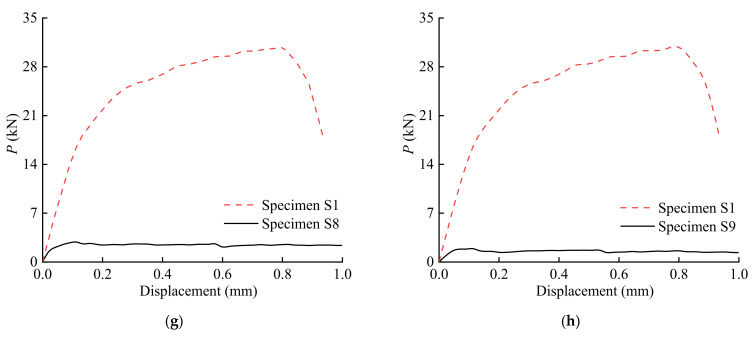
Load–displacement curves of specimens: (**a**) S2; (**b**) S3; (**c**) S4; (**d**) S5; (**e**) S6; (**f**) S7; (**g**) S8; (**h**) S9.

Figure 4 presents the load–displacement relationship of all the specimens. For the specimen without initial angles, the applied load increased to its ultimate value and then decreased until debonding failure. When the initial angle of the specimen was greater than 0°, the load increased to a certain peak value, and then decreased suddenly; afterward, it increased again until failure. The sudden decreases in the load represent the propagation of the delamination of the BFRP sheet caused by peeling. With an increase in the initial angle, the ultimate load of the specimen decreases gradually. Compared with the specimen under pure shear loading at the interface, the ultimate load decreased nearly 50% when the initial angle was 6°. The ultimate load of the specimen with an initial angle of 20° decreased about 94% compared with the base specimen. Therefore, the effect of the initial angle on the ultimate load of the specimen is significant.

### 3.3. Strain Distribution along the Bond Length

**Figure 5:**
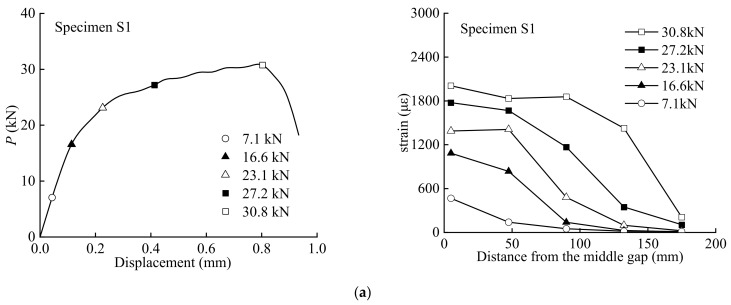

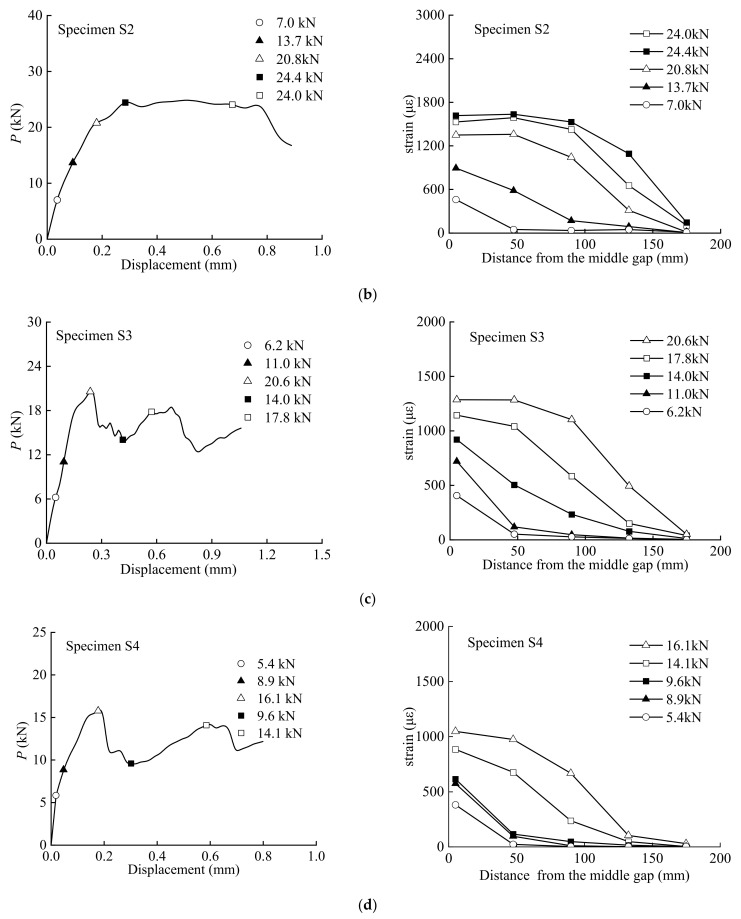

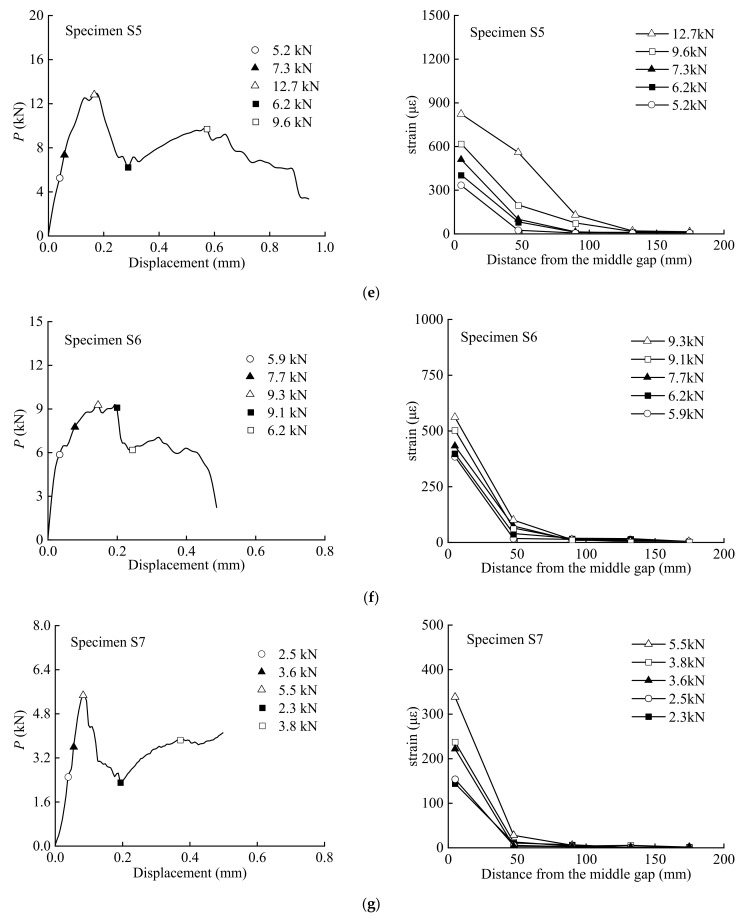

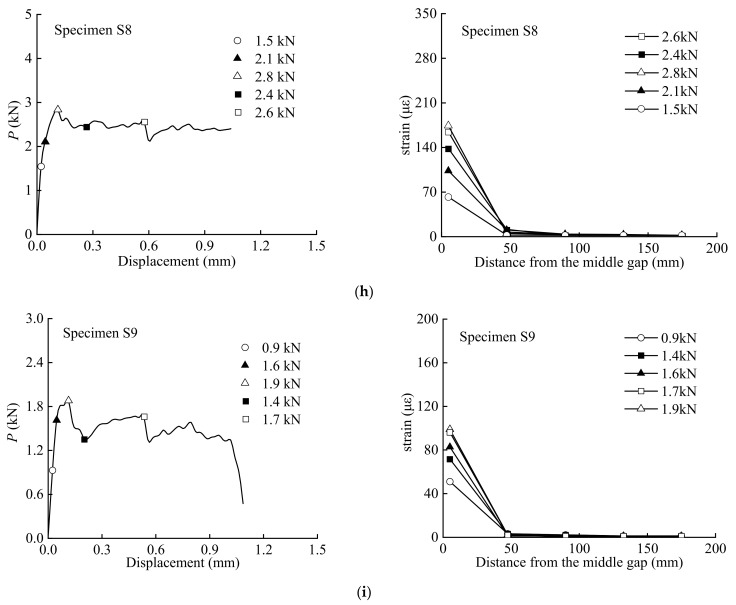
Strain distribution along BFRP sheet: (**a**) S1; (**b**) S2; (**c**) S3; (**d**) S4; (**e**) S5; (**f**) S6; (**g**) S7; (**h**) S8; (**i**) S9.

Figure 5 presents the strain distributions observed during the tests. The strain distributions corresponding to the marked points on the load–displacement curves are plotted. As can be seen, when the load was initially applied, the strain response was first observed only at the middle gap of the specimen, and the value increased with increasing load. Macroscopic debonding from the middle gap occurred when the maximum load was reached. When the initial angle was less than 6°, strain increased at the loaded end. As the strain approached the loaded end, a strain plateau was formed. The results from the tests show that the strain generally increased with the load levels and decreased from the gap at the middle of the specimen to the free end of the BFRP sheet. The debonding propagation of the interface can be observed from the strain distribution since there was no strain response in the undamaged regions.

## 4. Evaluation of Bond Strength

In order to determine the bond strength of the interface between the FRP sheets and the steel without an initial angle, several models have been proposed in the literature [22,28,44,45]. He et al. [22] and Lu et al. [45] proposed a model for calculating the bond shear strength of the CFRP-plate–steel interfaces using an effective bond length, *L*_e_. Liu [44] proposed an FRP–steel bond shear capacity model based on the double-lap joint tensile shear tests. Using these selected models, the experimental bond strength was normalized by the calculated bond strength to investigate the effect of the initial angle. The Liu model [44] was selected and used in the present study. The calculation formulae are expressed as follows:

(1) $$L_{e}=\sqrt{\frac{E_{p}t_{\mathrm{pm}}}{0.5f_{v}}}$$

(2) $$t_{\mathrm{pm}}=(1-\frac{E_{p}t_{p}}{420000})t_{p}$$

(3) $$P_{\mathrm{ult}}=\beta_{p}\beta_{a}f_{v}b_{p}L_{e}$$

(4) $$\beta_{p}=\sqrt{\frac{2-b_{p}/b_{s}}{1+b_{p}/b_{s}}}$$

(5) $$\beta_{a}=\left\{\begin{array}{l} 1\;,\;l\geq L_{e}\\ \sin\frac{\pi l}{2L_{e}},\;l<L_{e} \end{array}\right.$$

where *L*_e_ is the effective bond length, *P*_ult_ is the ultimate load, *b*_p_ is the width of the BFRP sheet, *b*_s_ is the width of the sheet plate, *t*_p_ is the thickness of bonded BFRP sheet, *t*_pm_ is the effective thickness of the bonded FRP sheet, *E*_p_ is the FRP laminate elastic modulus, *β*_p_ and *β*_a_ are dimensionless coefficients that reflect the effects of the FRP-to-steel width ratio *b*_p_/*b*_c_ and the bond length *l*; and *f*_v_ is the steel-to-steel shearing bonding strength of the adhesive.

**Figure 6:**
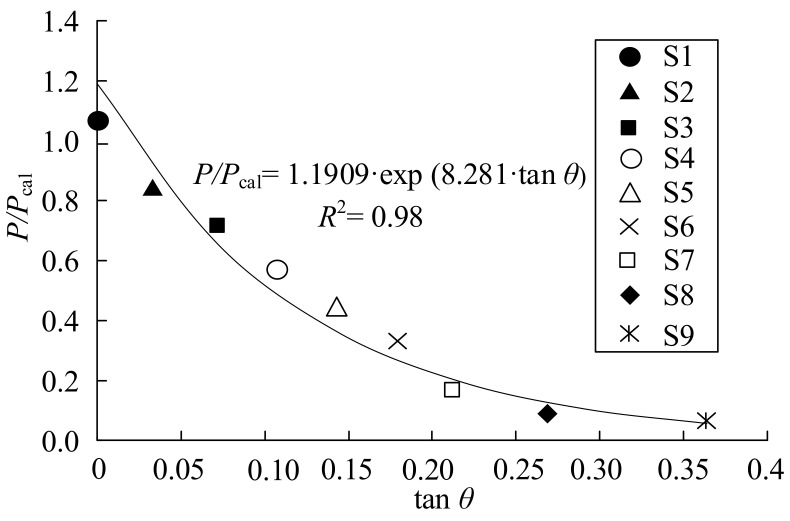
Effect of peeling angle on bond strength.

The relation between the tensile load *P* and the calculated tensile load *P*_cal_ based on the Liu model is plotted against the peeling angle (tan *θ*), as shown in Figure 6. It is evident that the tensile load decreases as the peeling angle increases. The relationship between the tensile load ratio and peeling angle can be obtained based on the experimental results by the following equation:

(6) $$P/P_{\mathrm{cal}}=1.1909\cdot \exp\left(-8.281\cdot \tan\theta \right)$$

**Figure 7:**
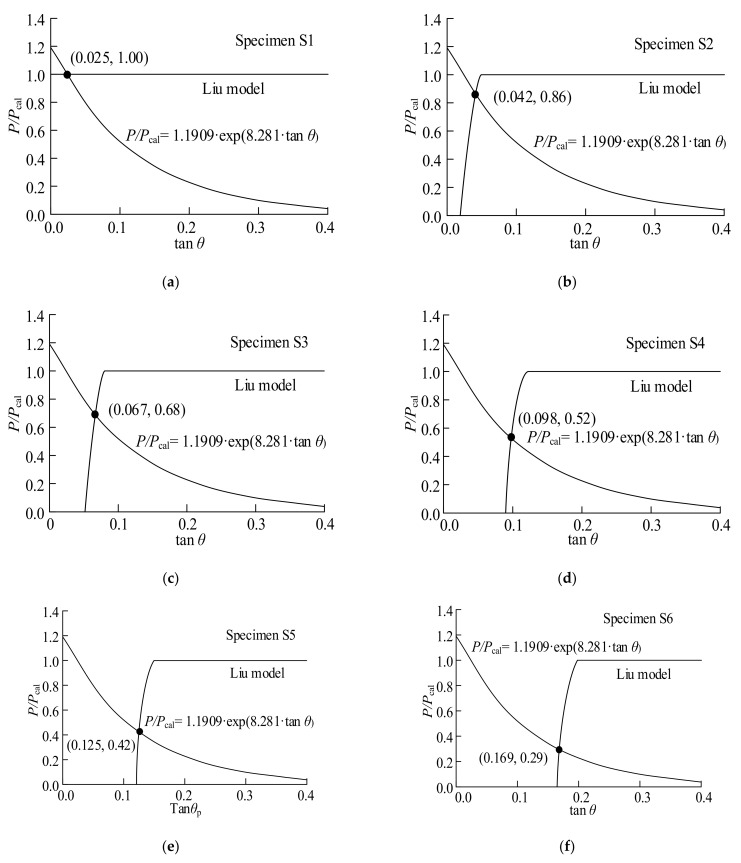

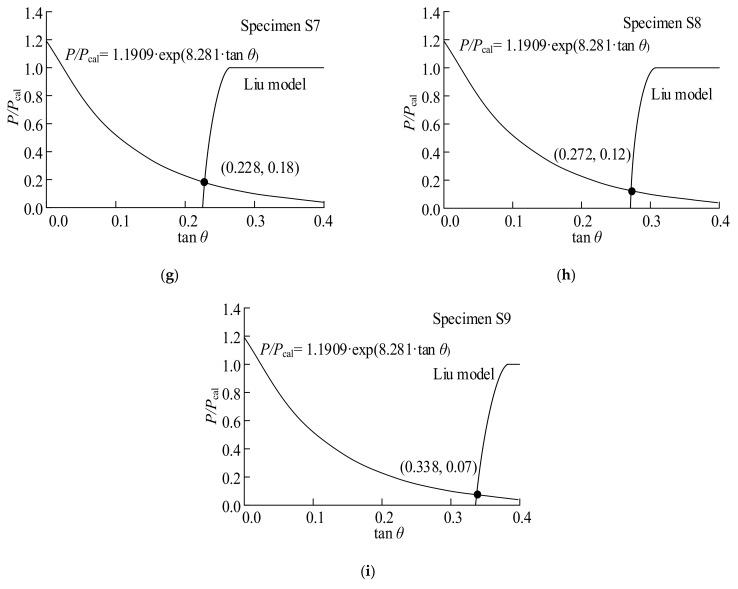
Evaluation of combined fracture bond strength for specimens: (**a**) S1; (**b**) S2; (**c**) S3; (**d**) S4; (**e**) S5; (**f**) S6; (**g**) S7; (**h**) S8; (**i**) S9.

The relationship between the tensile load and peeling angle was studied to evaluate the mixed-mode shear-peeling bond strength. The sheet delaminated as the load proceeded, resulting in a decrease in both the bond length and the peeling angle between the BFRP sheet and the steel surface. The delaminated region is defined as the sheet from the beginning point of the pre-unbond region to the position with a sudden decrease in strain. For example, in specimen S3, the values were determined based on the debonding region of the step height at which the strain gauge was located 43.5 mm from the specimen center when the first sudden drop occurred. Following that, the values were selected step by step, starting at the debonding region and extending to the strain gauge at 86.0 mm. The same method was used for selecting values in the debonding region, which extended to the strain gauge at 128.5 mm and 171.0 mm from the specimen center. Figure 7 illustrates the plots of the proposed equation and the predicted results of the Liu model, which reveals the trends in the shear bond strength curves calculated using the method described above. Experimental results show that the bond length decreased as the BFRP sheet delaminated. As a result, the bond length is shorter than the effective bond length.

**Figure 8:**
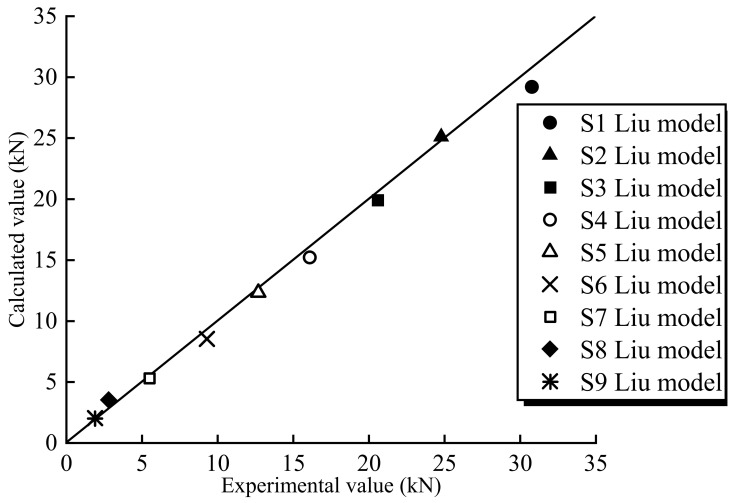
Relationship between experiment value *P* and calculated value *P*_cal_.

For the combined fracture mode, the intersection points in Figure 7 represent the mixed-mode bond strength. Therefore, the corresponding values of *P*/*P*_cal_ can be calculated using the values of tan *θ* at these intersections. The results are represented in Figure 8. The values of *P*/*P*_cal_ and tan *θ* are provided in Table 6. For all specimens, the analytical strengths are in good agreement with the experimental results.

## 5. Conclusions

The failure mode and debonding behavior of the interfaces between the basalt fiber reinforced polymer (BFRP) sheet and structural steel under mixed-mode loading conditions were experimentally studied. The effect of initial angles on the interfacial bond strength was investigated. A predicted method of the shear-peeling bond strength was proposed. Some conclusions can be drawn as follows.

(1) For the specimen without an initial angle, the applied load increased to its ultimate value and then decreased until debonding failure. When the initial angle of the specimen was greater than 0°, the load increased to a certain peak value and then decreased suddenly. Afterward, it increased again until failure.

(2) The specimens with a smaller initial angle were more likely to exhibit a shear debonding failure at the interface between the steel plate and adhesive. In contrast, the specimens with a larger initial angle were more likely to exhibit peeling of the interface.

(3) The ultimate tensile load of the specimen decreased with an increase in the initial angle. The initial angle had a significant influence on the interfacial bonding properties.

(4) A method to predict the interfacial bond strength under mixed-mode loading conditions was proposed. According to the proposed method, the bond strength between the BFRP sheet and the steel under combined shear and peeling fracture conditions can be accurately predicted.

## Figures and Tables

**Table 1:** Parameters of test specimens.

Specimen	S1	S2	S3	S4	S5	S6	S7	S8	S9
Initial angle *θ*	0°	2°	4°	6°	8°	10°	12°	15°	20°
Pre-unbond length (mm)	0.0	143.2	71.5	45.6	35.6	28.4	23.5	18.7	13.7

For each load case, 2 replicates were produced and tested.

**Table 2:** Material properties of adhesive.

Tensile Strength (MPa)	Flexural Strength (MPa)	Elongation at Break (%)	Compressive Strength (MPa)	Steel-to-Steel Joint (MPa)	Elastic Modulus (MPa)
35.0	45.0	1.4	67.0	17.3	3010.0

**Table 3:** BFRP sheet properties.

Thickness (mm)	Elastic Modulus (GPa)	Fiber Diameter (μm)	Tensile Strength (MPa)
0.2	85.0	8.0	2400.0

**Table 4:** Steel properties.

Steel Type	Thickness (mm)	Yield Strength (MPa)	Ultimate Strength (MPa)	Elastic Modulus (GPa)
Q345qD	10 and 20	397.5	585.4	210.0

**Table 5:** Test results of the specimens.

Specimen	Initial Angle *θ*	Ultimate Load *P*(kN)	Liu Model	*P*/*P*_cal_ (kN)
			Calculated Ultimate Load *P*_cal_ (kN)	
S1	0°	30.8	29.2	1.05
S2	2°	24.8		0.86
S3	4°	20.6		0.68
S4	6°	16.1		0.52
S5	8°	12.7		0.42
S6	10°	9.3		0.29
S7	12°	5.5		0.18
S8	15°	2.8		0.12
S9	20°	1.9		0.07

**Table 6:** Tested and predicted results.

Specimen	Initial Angle *θ*	Experimental Value *P* (kN)	tan *θ*	*P*/*P*_cal_		Predicted Value *P* (kN)	
				Equation (6)	Liu Model	Equation (6)	Liu Model
S1	0°	30.8	0.025	1.19	1.05	34.8	29.2
S2	2°	24.8	0.042	0.89	0.86	26.0	25.1
S3	4°	20.6	0.067	0.67	0.68	19.5	19.9
S4	6°	16.1	0.098	0.50	0.52	14.6	15.2
S5	8°	12.7	0.125	0.37	0.42	10.9	12.3
S6	10°	9.3	0.169	0.28	0.29	8.1	8.5
S7	12°	5.5	0.228	0.20	0.18	6.0	5.3
S8	15°	2.8	0.272	0.13	0.12	3.8	3.5
S9	20°	1.9	0.338	0.06	0.07	1.7	2.0

## Data Availability

Data is contained within the article.
